# Speaking and Singing without a Tongue

**Published:** 1871-02

**Authors:** 


### SPEAKING AND SINGING WITHOUT A TONGUE.

In the transactions of the Philosophical Society, published between 1742 and 1744, there is an account of Margaret Cutter, who when four years old, lost her entire tongue from a cancerous affection; but who, nevertheless, afterward retained the power of taste, swallowing, and speech, without any imperfection whatever. She not only spoke as fluently and with as much correctness as other people, but also sung to admiration, articulating with distinctness all her words while singing What is not less singular, she could form no idea of the use of a tongue in other persons. This remarkable case was brought before the Royal Society, under certificates of attestation from the minister of the parish, a medical practitioner, and another respectable citizen, well-known in Suffolk, where she resided. On account of the extraordinary character of the case, the Society requested an additional report on the subject, and from another set of witnesses, named by the Society for the purpose, and for whom they drew up the necessary questions and marked out the proper course of examination. The second report coincided with the first in all particulars, and shortly afterward the young woman was brought to London, where she confirmed the account by personally appearing, and speaking and singing in the presence of the members of the Royal Society and many other persons.—*The College Courant.*

